# Investigating the Clinico-Molecular and Immunological Evolution of Lung Adenocarcinoma Using Pseudotime Analysis

**DOI:** 10.3389/fonc.2022.828505

**Published:** 2022-03-04

**Authors:** Hyunjong Lee, Hongyoon Choi

**Affiliations:** ^1^ Department of Nuclear Medicine, Samsung Medical Center, Sungkyunkwan University School of Medicine, Seoul, South Korea; ^2^ Department of Molecular Medicine and Biopharmaceutical Sciences, Graduate School of Convergence Science and Technology, Seoul National University, Seoul, South Korea; ^3^ Department of Nuclear Medicine, Seoul National University College of Medicine, Seoul, South Korea

**Keywords:** lung adenocarcinoma, stage, glucose metabolism, tumor immune microenvironment, pseudotime analysis

## Abstract

**Introduction:**

As the molecular features of lung adenocarcinoma (LUAD) have been evaluated as a cross-sectional study, the course of tumor characteristics has not been modeled. The temporal evolution of the tumor immune microenvironment (TIME), as well as the clinico-molecular features of LUAD, could provide a precise strategy for immunotherapy and surrogate biomarkers for the course of LUAD.

**Methods:**

A pseudotime trajectory was constructed in patients with LUAD from the Cancer Genome Atlas and non-small cell lung cancer radiogenomics datasets. Correlation analyses were performed between clinical features and pseudotime. Genes associated with pseudotime were selected, and gene ontology analysis was performed. F-18 fluorodeoxyglucose positron emission tomography images of subjects were collected, and imaging parameters, including standardized uptake value (SUV), were obtained. Correlation analyses were performed between imaging parameters and pseudotime. Correlation analyses were performed between the enrichment scores of various immune cell types and pseudotime. In addition, correlation analyses were performed between the expression of PD-L1, tumor mutation burden, and pseudotime.

**Results:**

Pseudotime trajectories of LUAD corresponded to clinical stages. Molecular profiles related to cell division and natural killer cell activity were changed along the pseudotime. The maximal SUV of LUAD tumors showed a positive correlation with pseudotime. Type 1 helper T (Th1) cells showed a positive correlation, whereas M2 macrophages showed a negative correlation with pseudotime. PD-L1 expression showed a negative correlation, whereas tumor mutation burden showed a positive correlation with pseudotime.

**Conclusion:**

The estimated pseudotime associated with the stage suggested that it could reflect the clinico-molecular evolution of LUAD. Specific immune cell types in the TIME as well as cell division and glucose metabolism were dynamically changed according to the progression of the pseudotime. As a molecular progression of LUAD, different cellular targets should be considered for immunotherapy.

## Introduction

Lung adenocarcinoma (LUAD) is the most frequent histological type among lung malignancies (1). The diagnosis and treatment of LUAD are based on initial evaluation of disease progression. TNM stage has been recognized as the most basic and critical factor to evaluate the status of disease (2). In addition, the progression of lung cancer has been assessed by histological features and imaging findings as well as staging. Among imaging modalities, ^18^F-fluorodeoxyglucose positron emission tomography (FDG PET) is widely used to evaluate the extent of metastasis and aggressiveness of tumors (3, 4). However, there is a limitation to investigating the natural progression of tumors based on conventional diagnostic information, as pathological staging and imaging examinations are performed at the timing of initial diagnosis. Therefore, the biological and molecular progression of LUAD has hardly been modeled on a continuous scale.

The tumor immune microenvironment (TIME) plays a crucial role in tumor progression and metastasis. Because of recent broad-range indications for immune checkpoint inhibitors, tumor immune profiles in addition to staging have been suggested for predicting prognosis and considering appropriate treatment plans (5). Among immune cells, natural killer (NK) cells and T cells are known to have a role in antitumor immunity (6, 7). In addition, tumor-associated macrophages (TAMs) exert various functions in lung cancer by differentiating into different subtypes: M1 and M2 macrophages, with M1 macrophages mainly contributing to antitumor activity, and M2 macrophages mainly contributing to protumor activity (8). In the clinical aspect, characterization of the TIME is important to explore therapeutic targets and predict the response to immunotherapy (9, 10). Therefore, it is important to investigate the evolution of the TIME during disease progression. More specifically, recent trends in immunotherapy suggest a strategy according to the characteristics and the progression pattern of the TIME (11).

Pseudotime analysis, also called trajectory inference analysis, is a spotlighted method to explore changes in cell or tissue characteristics based on transcriptomic expression (12). It provides a numerical scale to reflect where a cell or tissue is in the course of disease, other than the TNM staging system. Although there have been several studies to apply pseudotime analysis in lung cancer samples, the scopes of those studies were limited to only single-cell RNA-sequencing (RNA-seq) data from small numbers of patients (13, 14). Pseudotime analysis in large numbers of subjects may provide a model to explore the course of biological progression of lung cancer.

In this study, we aimed to reveal the evolution of the TIME along with the molecular progression of LUAD. Pseudotime trajectories were estimated in the LUAD cohorts from The Cancer Genome Atlas (TCGA) and a non-small cell lung cancer (NSCLC) radiogenomics dataset. Associations between TIME cell types as well as clinico-molecular features and pseudotime were analyzed. We expected to find appropriate targets of the TIME according to the evolution of the TIME along the pseudotime.

## Methods

### Pseudotime Estimation

A pseudotime trajectory was constructed based on the sum of two publicly available datasets: TCGA-LUAD and TCGA-lung squamous cell carcinoma (LUSC). The datasets were obtained using the “TCGAbiolinks” package in R (15). Legacy data of gene expression quantification were downloaded using the “GDCdownload” function. There were 600 LUAD samples and 553 LUSC samples. RNA-seq data were prepared as a matrix format and normalized by log2 transformation. Highly variable genes were selected using the “DESeq2” package in R (16, 17). First, variance and coefficients of variation for each gene expression were calculated in 1153 total samples. Subsequently, a generalized linear model was fitted to set a reference for variability of each gene expression using “glmgam.fit” function in R. The fitted curve was hypothesized as an expected distribution of estimates of variance and coefficients of variation. Chi-squared tests were performed to evaluate deviation from the fitted curve. Finally, genes showing lower p-value than 0.001 were selected as highly variable genes (HVGs). A total of 8589 genes were selected among a total of 21,022 genes. A pseudotime trajectory was generated using the “Phenopath” package in R (18). PhenoPath, an analytic tool for pseudotime, provides an ordering of gene expression measurements across individual objects. It employs a Bayesian statistics and models latent progression of gene expression (18). Among various pseudotime analysis tools, PhenoPath was the only method to be utilized in bulk tissue RNA-seq dataset. Therefore, it was selected in the present study. The input data were a gene expression matrix of HVGs from TCGA-LUAD and TCGA-LUSC datasets. We chose the evidence lower bound as 10^-6^ and computed it thinned by 2 iterations. Ultimately, pseudotime as a reference value for latent progression of disease was estimated.

### Applying Pseudotime Into a New Dataset

LUAD samples of the NSCLC radiogenomics dataset were employed to perform additional correlative analysis with imaging-derived variables. An RNA-seq dataset (GSE103584) was downloaded from the Gene Expression Omnibus (https://www.ncbi.nlm.nih.gov/geo/) (19). There were 96 LUAD samples in the NSCLC radiogenomics dataset. To translate pseudotime estimated by the TCGA dataset to the new dataset, a lasso regression model was used to estimate pseudotime in LUAD samples of the NSCLC radiogenomics dataset. There were three reasons to apply lasso regression model. First, there was a technical limitation that ‘Phenopath’ tool could not generate a proper model due to small sample size of the NSCLC radiogenomics dataset. Second, it was necessary to predict pseudotime based on genes which were revealed to have significant association with pseudotime in TCGA dataset. Third, lasso regression provides better interpretability and prevents overfitting as it deals some of the estimated coefficients as zero (20). To develop a model to predict pseudotime using a lasso regression, two hundred genes were selected from genes that showed a significant association between pseudotime: the top 100 genes with highest correlation coefficients in the positive correlation group and the top 100 genes with highest correlation coefficients in the negative correlation group. An expression matrix of those 200 genes was constructed from the TCGA dataset. It was divided into two groups at a 2:1 ratio: training and internal validation data. The lambda with the least error was selected *via* cross-validation. A lasso regression model was obtained. The alpha was 1. The model was applied to LUAD samples of the NSCLC radiogenomics dataset to predict a pseudotime trajectory.

### Clinical Feature Analysis

Clinical data of the TCGA dataset were downloaded from cBioPortal (http://www.cbioportal.org/) using the “cgdsr” package in R. TNM stage, disease-free survival (DFS), overall survival (OS), and duration of DFS/OS were selected as representative clinical factors. A heatmap was plotted to visualize the associations between genes, clinical factors, and pseudotime using the “Complexheatmap” package in R. The pseudotimes of each TNM stage group were compared using t-tests or ANOVA. Survival analyses for DFS and OS were performed using the Kaplan–Meier method between subjects with early and late pseudotime. Cutoff values of pseudotime were explored using the “cutoff” package in R. Clinical data of the NSCLC radiogenomics dataset were downloaded from The Cancer Imaging Archive (TCIA, https://www.cancerimagingarchive.net/). The pseudotime of each TNM stage group was compared using the Wilcoxon rank-sum test or the Kruskal–Wallis test.

### Genetic Feature Analysis

Principal component analysis (PCA) was performed to visualize the temporal evolution of the genetic characteristics of LUAD and LUSC using the “PCA” function included in the “factoextra” package in R. Phenopath analysis provided four output values: *alpha*: degree of differential expression, *beta*: degree of covariate-pseudotime interaction, *lambda*: degree of pseudotime dependency, *z*: estimates of pseudotime. A Bayesian significance test was applied to select genes showing significant pseudotime dependency (nonzero lambda) and significant covariate pseudotime dependency (nonzero beta). Gene ontology (GO) analysis was conducted on genes showing significant pseudotime dependency to investigate which functions were upregulated or downregulated along the pseudotime trajectory using the “enrichGO” function included in the “clusterProfiler” package in R. A cut-off of p-value was 0.05 and that of q-value was 0.1 to select significant GO terms.

### Glucose Metabolism Analysis

FDG PET images of LUAD subjects from both the TCGA dataset and the NSCLC radiogenomics dataset were downloaded from The Cancer Imaging Archive (TCIA, https://www.cancerimagingarchive.net/). There were 16 and 93 samples with both RNA-seq data and FDG PET images in LUAD samples of the TCGA and NSCLC radiogenomics datasets, respectively. Tumor margins were delineated using the Nestle adaptive threshold method provided by “LifeX” software (21, 22). An adaptive threshold to define tumor margins was applied, and the deterministic parameter *beta* was set to 0.3. The maximal standardized uptake value (SUV), mean SUV, and metabolic tumor volume (MTV) were obtained from the region of interest. Total lesion glycolysis (TLG) was calculated from the mean SUV and MTV. Correlation coefficients of expression of FDG PET parameters, solute carrier family 2 member 1 (SLC2A1) expression, and pseudotime were calculated by Spearman and Pearson correlation tests in the TCGA and NSCLC radiogenomics datasets, respectively.

### Immune Profile Analysis

In both the TCGA and NSCLC radiogenomics datasets, the enrichment scores of 64 immune and stromal cell types were estimated using the “xCellAnalysis” function in the “xCell” package in R (23). Correlation coefficients between enrichment scores and pseudotime were calculated by the Pearson correlation test. The false discovery rate was calculated from p values with the Bonferroni method. Volcano plots, heatmaps, and scatter plots were generated to describe the association between the enrichment scores of immune cells and pseudotime. The expression of PD-L1 and the tumor mutation burden (TMB) are well-known indicators of the immune profiles of tumors (24, 25). In the TCGA dataset, gene mutation data were downloaded from genomic data commons (https://gdc.cancer.gov/), and a mutation annotation format file was then constructed using the “read.maf” function included in the “maftools” package (26). TMB was calculated by the number of non-synonymous somatic mutations using the “tmb” function included in the “maftools” package. Correlation coefficients of expression of PD-L1 and TMB with pseudotime were calculated by the Pearson correlation test. All statistical analyses were performed using R software (v4.0.4, Vienna, Austria). A p value of < 0.05 was considered statistically significant.

## Results

### Clinical Features Related to Pseudotime

**Figure 1:**
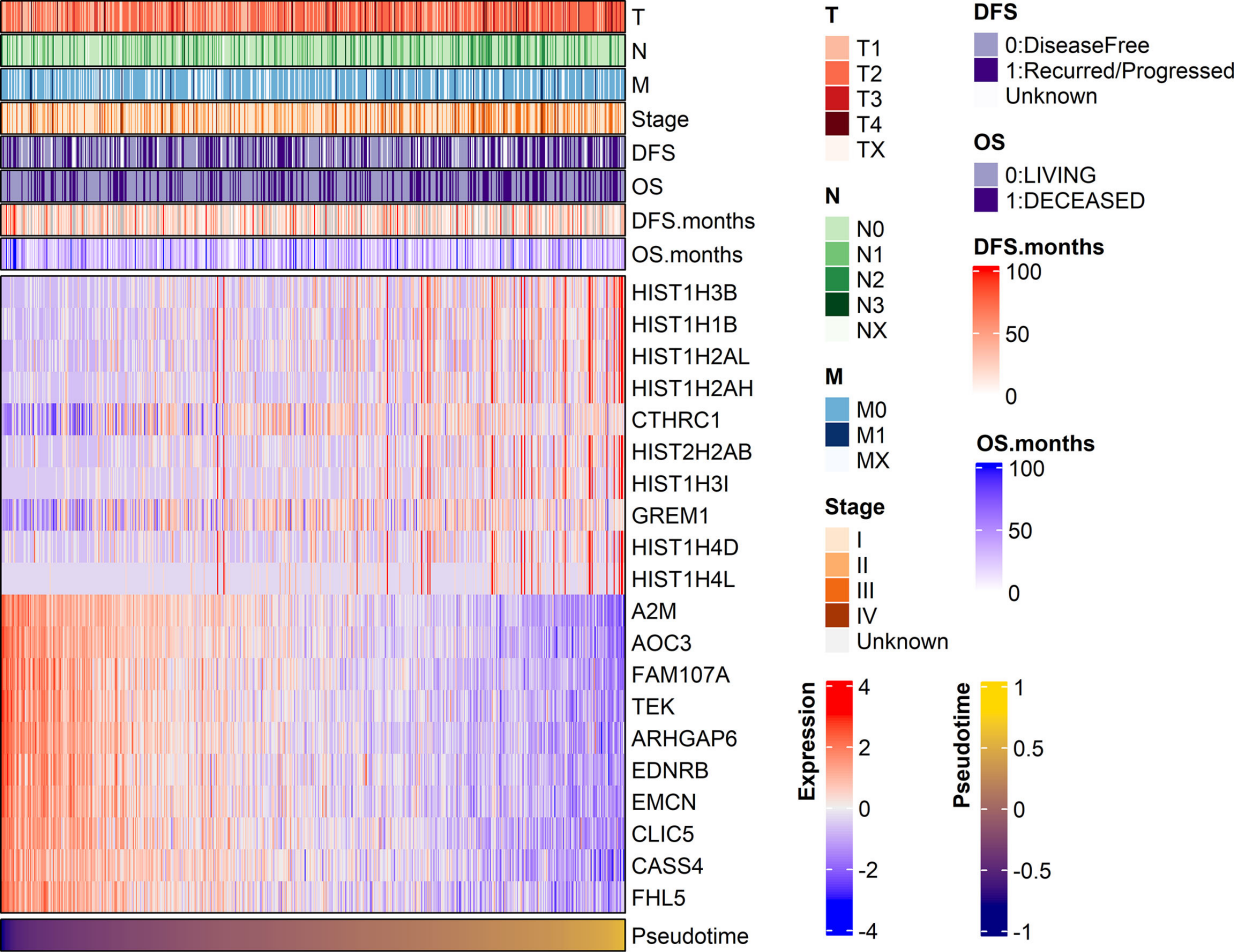
A heatmap visualizing clinical features and gene expression along pseudotime in the TCGA-LUAD dataset. Clinical features, including TNM stage and the expression of the top 10 genes showing significant positive or negative associations with pseudotime, are displayed. In particular, histone-coding genes showed significant upregulation over pseudotime.

**Figure 2:**
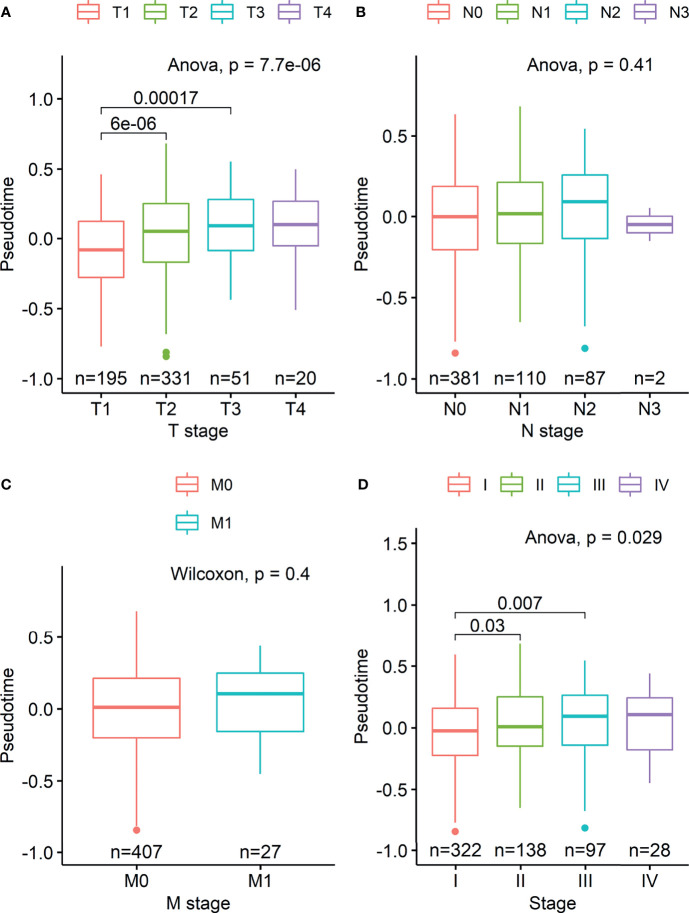
Boxplots visualizing differences in pseudotime according to TNM stage in the TCGA-LUAD dataset. **(A)** There was a significant difference in pseudotime in each T stage, especially in T1-T2 (mean: -0.08013 vs. 0.03092, p < 0.001) and T1-T3 (mean: -0.08013 vs. 0.08310, p < 0.001). **(B, C)** Pseudotime in each N and M stage showed no difference. **(D)** There was a significant difference in pseudotime in each overall TNM stage, especially in IA-IB (mean: -0.09326 vs. 0.02969, p < 0.001), IA-IIB (mean: -0.09326 vs. 0.05374, p < 0.001), and IA-IIIA (mean: -0.09326 vs. 0.03749, p < 0.001).

**Figure 3:**
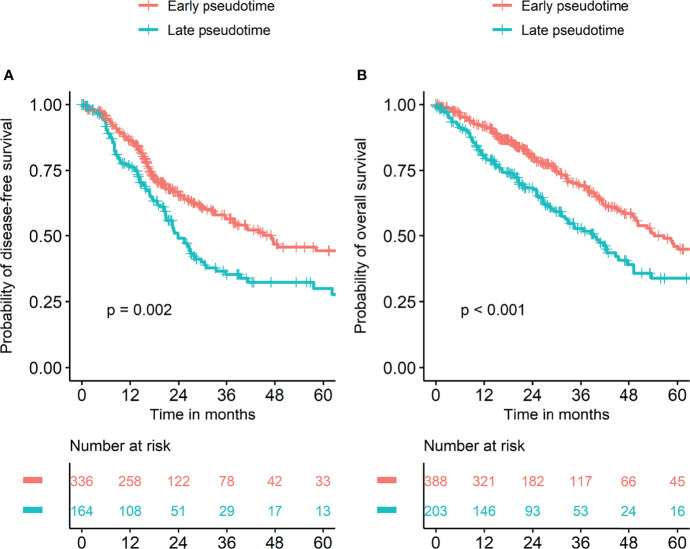
Survival curves according to pseudotime. Survival analyses were performed in two groups divided by cutoff values of pseudotime which were explored using the “cutoff” package in R. **(A)** Disease-free survival was significantly different between the two groups. **(B)** Overall survival was significantly different between the two groups.

Demographic and clinical characteristics of the patients were described in **Table 1**. A heatmap was constructed to visualize the clinical factors of each sample with the top 10 genes upregulated and those downregulated over pseudotime in TCGA-LUAD samples (**Figure 1**). Notably, histone coding genes showed upregulation along pseudotime. Boxplots represent the association between TNM stage and pseudotime in LUAD samples (**Figures 2A–D**). There was a significant difference in pseudotime in each T stage (p < 0.001), especially in T1-T2 (mean: -0.08013 vs. 0.03092, p < 0.001) and T1-T3 (mean: -0.08013 vs. 0.08310, p < 0.001). Pseudotime in different N stages and M stages showed no difference. There was a significant difference in pseudotime in each overall TNM stage (p < 0.001), especially in I-II (mean: -0.02835 vs. 0.03565, p = 0.021) and I-III (mean: -0.02835 vs. 0.04780, p = 0.019). Disease-free survival and overall survival were well discriminated according to pseudotime (**Figure 3**, p = 0.002 and p < 0.001, respectively).

**Table 1:** Demographic and clinical characteristics of the patients.

Characteristics	TCGA-LUAD	LUAD in the NSCLC radiogenomics dataset
	Patients, n (%)	Patients, n (%)
Total	600	96
Sex		
Female	325 (54.2)	29 (30.2)
Male	275 (45.8)	67 (69.8)
Age, median (range), years	66 (33-88)	68 (43-85)
Smoking history		
Current	131 (21.8)	19 (19.8)
Former	364 (60.7)	57 (59.4)
Never	85 (14.2)	20 (20.8)
Unknown	20 (3.3)	0
Location		
Right upper lobe	220 (36.7)	31 (32.3)
Right middle lobe	24 (4)	8 (8.3)
Right lower lobe	106 (17.7)	12 (12.5)
Left upper lobe	144 (24)	30 (31.3)
Left lower lobe	88 (14.7)	15 (15.6)
Unknown	18 (3)	0
Pathological T stage		
Tis	0	5 (5.2)
T1	195 (32.5)	38 (39.6)
T2	331 (55.2)	38 (39.6)
T3	51 (8.5)	11 (11.5)
T4	20 (3.3)	4 (4.2)
Unknown	3 (0.5)	0
Pathological N stage		
N0	381 (63.5)	78 (81.3)
N1	110 (18.3)	7 (7.3)
N2	87 (14.5)	11 (11.5)
N3	2 (0.3)	0
Unknown	20 (3.3)	0
Pathological M stage		
M0	407 (67.8)	91 (94.8)
M1	27 (4.5)	5 (5.2)
Unknown	166 (27.7)	0
Pathological stage		
0	0	5 (5.2)
I	322 (53.7)	57 (59.4)
II	138 (23)	16 (16.7)
III	97 (16.2)	13 (13.5)
IV	28 (4.7)	5 (5.2)
Unknown	15 (2.5)	0

Pseudotime was estimated in the NSCLC radiogenomics dataset based on a lasso regression model from the TCGA dataset. The association between clinical factors and pseudotime was evaluated in LUAD samples of the NSCLC radiogenomics dataset. There was a tendency of increasing T stage along pseudotime, especially in early T stages (**Supplementary Figure 2A**, p = 0.097). There was no association between N/M stage and pseudotime (**Supplementary Figures 2B, C**). Histological grade showed an association with pseudotime (**Supplementary Figure 2D**, p = 0.017). There was no significant association between overall TNM stage and pseudotime (**Supplementary Figure 2E**).

### Genetic and Functional Features Related to Pseudotime

**Figure 4:**
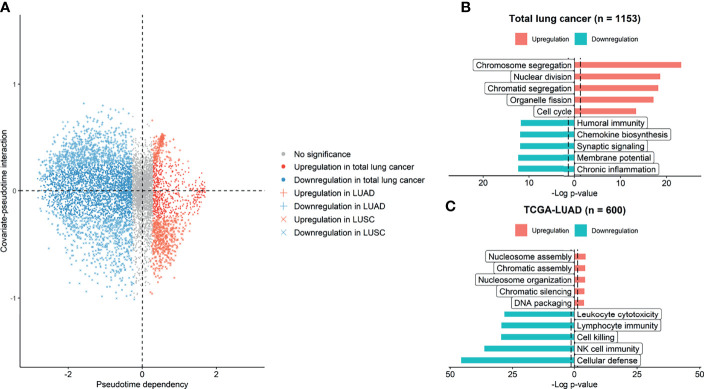
Genes and molecular functions upregulated and downregulated over pseudotime. **(A)** Genes upregulated and downregulated over pseudotime were selected and plotted. A total of 3197 genes showed significant correlations with pseudotime in total lung cancer. A total of 1031 genes showed significant correlations in LUAD samples, and 1126 genes showed significant correlations in LUSC samples. **(B)** In total lung cancer samples, molecular functions related to cell division were upregulated over pseudotime. **(C)** In LUAD samples, those related to natural killer cell activity were downregulated over pseudotime, and those related to cell division were upregulated.

We investigated gene expression features and their functional relevance according to pseudotime. On the dimension reduction plot using PCA, the LUAD and LUSC samples seemed to be in the same position at the beginning of pseudotime (**Supplementary Figure 3**). As pseudotime passed, the LUAD and LUSC samples were clearly discriminated in the PCA plot. We investigated genes regulated over pseudotime in total lung cancer, LUAD, and LUSC samples (**Figure 4A**). A total of 603 genes showed significantly positive correlations with pseudotime in total lung cancer samples, 2594 genes showed negative correlations in total cancer samples, 178 genes showed positive correlations in LUAD samples, 853 genes showed negative correlations in LUAD samples, 479 genes showed positive correlations in LUSC samples, and 647 genes showed negative correlations in LUSC samples. GO analysis was performed to determine which biological pathways were related (**Figures 4B, C**). In total lung cancer samples, molecular functions related to cell division were upregulated over pseudotime. In LUAD samples, molecular functions related to cell division, such as nucleosome assembly and DNA packaging, were upregulated over pseudotime, as in total lung cancer samples. Those related to NK cell function are downregulated over pseudotime.

### Evolution of Glucose Metabolism Along Pseudotime

**Figure 5:**
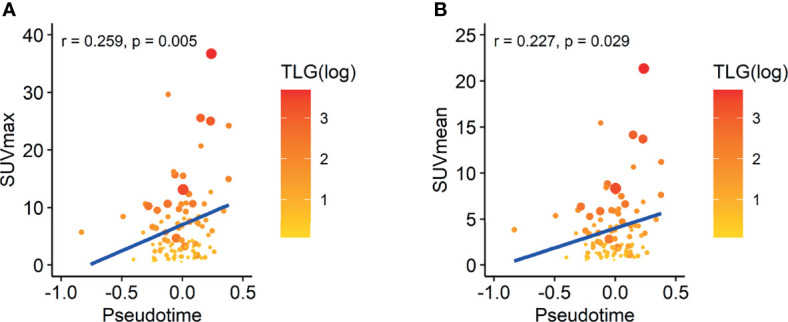
Correlation between SUV and pseudotime in LUAD samples of the NSCLC radiogenomics dataset. The size of the dot represents the metabolic tumor volume. The color of the dot represents total lesion glycolysis as a log scale. **(A)** The maximal SUV showed a weakly positive correlation with pseudotime (r = 0.259, p = 0.005). **(B)** The mean SUV showed a weakly positive correlation with pseudotime (r = 0.227, p = 0.029). However, MTV and TLG showed no association with pseudotime.

As increased glucose metabolism measured by FDG PET is associated with poor prognosis by reflecting biological aggressiveness, we tested the association between FDG uptake and pseudotime (27). In the TCGA-LUAD dataset, there was a significant positive correlation between maximal SUV and pseudotime (**Supplementary Figure 4**, rho = 0.518, p = 0.042). There was also a significant positive correlation between mean SUV and pseudotime (**Supplementary Figure 4**, rho = 0.517, p = 0.049). However, MTV and TLG showed no association with pseudotime. In the NSCLC radiogenomics dataset, there was a significant positive correlation between maximal SUV and pseudotime (**Figure 5A**, r = 0.259, p = 0.005). There was also a significant positive correlation between mean SUV and pseudotime (**Figure 5B**, r = 0.227, p = 0.029). However, MTV and TLG showed no association with pseudotime.

### Evolution of Immune Profiles Along Pseudotime

**Figure 6:**
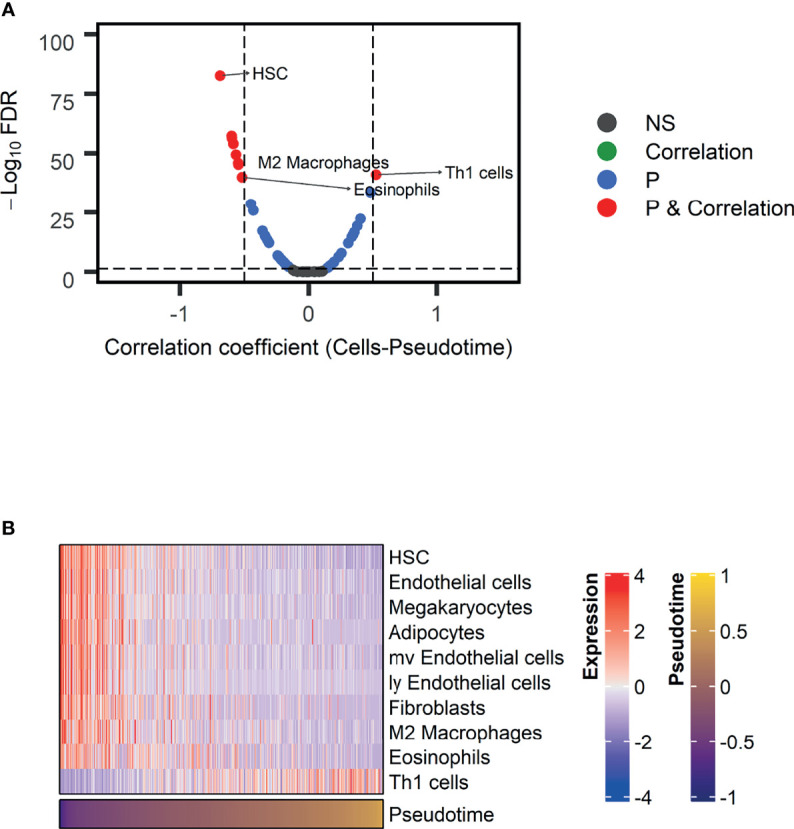
Immune and stromal cells associated with pseudotime in the TCGA-LUAD dataset. **(A)** A volcano plot representing immune cells associated with pseudotime is shown. Cell types with correlation coefficients above 0.5 are plotted as red dots. Cell types with FDR below 0.05 and correlation coefficients below 0.5 are plotted as blue dots. Among them, immune cells were annotated. **(B)** A heatmap representing immune and stromal cells associated with pseudotime is shown.

**Figure 7:**
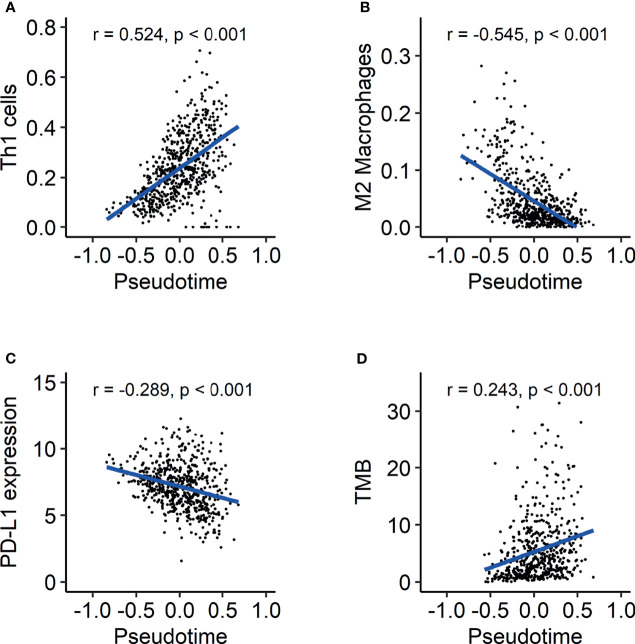
Evolution of the tumor immune microenvironment over pseudotime in the TCGA-LUAD dataset. **(A)** Th1 cells showed a positive correlation with pseudotime (r = 0.524, p < 0.001). **(B)** M2 macrophages showed a negative correlation with pseudotime (r = -0.545, p < 0.001). **(C)** PD-L1 expression showed a weakly negative correlation with pseudotime (r = -0.289, p < 0.001). **(D)** TMB showed a weakly positive correlation with pseudotime (r = 0.243, p < 0.001).

As GO terms related to pseudotime included downregulation of immune-related functions according to pseudotime, we further analyzed tumor immune microenvironment profiles related to pseudotime. A volcano plot and a heatmap were constructed to illustrate the immune and stromal cells associated with pseudotime in the TCGA dataset. (**Figures 6A, B**). Among cell types, the enrichment score of type 1 helper T (Th1) cells showed a positive correlation (**Figure 7A**, r = 0.524, p < 0.001), and that of M2 macrophages showed a negative correlation (**Figure 7B**, r = -0.545, p < 0.001). PD-L1, the most representative immunotherapy target in lung cancer, showed a weakly negative correlation with pseudotime (**Figure 7C**, r = -0.289, p < 0.001). TMB showed a weakly positive correlation with pseudotime (**Figure 7D**, r = 0.243, p < 0.001). In the NSCLC radiogenomics dataset, Th1 cells showed a positive correlation (**Supplementary Figure 5A**, r = 0.444, p < 0.001), and M2 macrophages showed a negative correlation (**Supplementary Figure 5B**, r = -0.367, p = 0.020). PD-L1 showed no significant correlation with pseudotime (**Supplementary Figure 5C**, r = 0.041, p = 0.698).

## Discussion

TNM staging in lung cancer is a well-established system to evaluate disease progression status, predict prognosis, and select appropriate treatment options (28, 29). However, it is the result of cross-sectional observation *via* clinical/pathologic/radiologic findings at the timing of initial diagnosis. Therefore, there is a limitation to investigating the temporal evolution of tumor biology longitudinally based on TNM staging as a reference scale. We attempted to construct a temporal model for biological progression and change of TIME from genetic profiles of a bulk RNA-seq dataset using pseudotime analysis. For scRNA-seq dataset, pseudotime analysis orders cells along a hypothetic trajectory based on patterns of gene expression (30). It is based on the hypothesis that multiple cross-sectional data is integrated into sequential data demonstrating temporal evolution (31). As bulk RNA-seq can be used for the trajectory analysis with a same manner, Campbell and Yau uncovered temporal evolution of tumor tissues of colorectal cancer and breast cancer (18). In this study, continuous scale for temporal evolution of tumor tissue was generated using previously known analytic tool, PhenoPath (18). The validity of the generated model as a reference of disease progression was examined by correlation analyses between clinico-molecular features and pseudotime. Based on the generated model, we investigated the temporal evolution of the tumor immune microenvironment in lung adenocarcinoma.

In this study, we successfully estimated a pseudotime trajectory in TCGA-LUAD and TCGA-LUSC datasets. In PCA, LUAD and LUSC samples in the early phase are revealed to have similar genetic characteristics and to differentiate into LUAD and LUSC along pseudotime order. In the tumorigenesis of NSCLC, molecular events such as 3p allele loss and telomerase activation are observed in most NSCLCs (32–34). The similarity of genetic characteristics in early LUAD and LUSC may be caused by common pathogenesis mechanisms. This result implies that tumors showing specific characteristics of LUAD or LUSC have a high possibility of progressed disease.

In both total lung cancer and LUAD samples, GO terms related to cell division were selected as significantly upregulated terms over pseudotime. In particular, histone-related genes showed a high correlation with pseudotime in LUAD. These results can be interpreted as the result of either the presence of a large number of tumor cells or enhanced mitotic activity of tumor cells in the late phase. Similarly, T stage and overall TNM stage demonstrated good association with pseudotime. This is consistent with the current TNM staging system. Notably, there were significant associations in the early T stages (T1-T2, T2-T3) and early overall stages (IA-IB, IA-IIB, IA-IIIA). In the current TNM staging system, T2-T4 stages include not only size criteria but also criteria involving other structures, such as bronchi or chest walls (35). Thus, a small tumor with involvement of other structures can be diagnosed as a high T stage. If there is lymph node metastasis, it is highly likely to be classified as above stage IIIA. These characteristics of the current TNM staging system cause associations between early T stage/overall stage and pseudotime. The probability of disease-free survival and overall survival showed significant differences according to pseudotime. This indicates that pseudotime may have clinical usability to classify patients based on prognosis as TNM staging. Of course, further study is warranted to explore the clinical significance of pseudotime trajectories.

In both datasets, the maximal SUV and mean SUV demonstrated significantly positive correlations with pseudotime. These findings are consistent with a previously revealed relationship between FDG uptake and tumor stage (3, 4, 36). Furthermore, the tendency of increasing FDG uptake along pseudotime is closely related to the molecular function of cell division, showing the same tendency along pseudotime. Proliferative activity is known to be a significant factor affecting FDG uptake in tumors in lung cancer (37, 38). In brief, changes in genetic features, clinical features, and glucose metabolism over pseudotime were revealed to be consistent with previous knowledge about tumor progression. Therefore, the estimated pseudotime was hypothesized to be an appropriate temporal reference of disease progression.

The present study demonstrated the temporal evolution of immune profiles in LUAD. It is noteworthy that the enrichment score of Th1 cells represented a significantly positive correlation with pseudotime. It is generally believed that Th1 cells contribute to the antitumor response, inducing cytotoxicity (7, 39). It is also remarkable that M2 macrophages showed a significantly negative correlation with pseudotime. M2 macrophages exert protumor activity *via* tissue remodeling and angiogenesis (40, 41). Briefly, antitumor immunity seems to strengthen along pseudotime, whereas protumor immunity seems to weaken along pseudotime. These results indicate two possible mechanisms. First, protumor immunity *via* M2 macrophages prepares an appropriate environment for the survival and proliferation of tumor cells in the early phase of lung cancer. Second, antitumor immunity *via* Th1 cells is induced by a high tumor burden to attempt to control and suppress disease in the late phase of lung cancer. These results are also consistent with a previous report documenting that the proportion of high stage was larger in samples with high immune scores and cytolytic scores (42). The present study showed heterogeneous evolution of specific immune cells over pseudotime. This implies that selecting immunotherapy options for appropriate targets may be considered during the disease progression of LUAD. Pharmaceuticals such as resveratrol and imatinib were found to suppress cancer progression *via* inhibition of M2 macrophage activation (43, 44). The application of those drugs may be utilized in patients with early pseudotime.

Interestingly, PD-L1 expression showed a negative correlation with pseudotime, whereas TMB showed a positive correlation in the TCGA dataset. These are well-known biomarkers predicting the response to cancer immunotherapy (25, 45). This implies that the response to immunotherapy, such as pembrolizumab, may not represent any tendency according to the molecular progression of lung adenocarcinoma. Referring to this heterogeneous finding of opposite tendencies of PD-L1 and TMB, it is necessary to establish a more precise immunotherapy strategy. Considering that both the enrichment of Th1 cells and TMB showed positive correlations with pseudotime, it is supposed that immunogenic antigens are enriched in progressed LUAD. LUAD with a relatively early pseudotime associated with high PD-L1 suggested early anti-PD-1/PD-L1 treatment before tumor evolution (46). As LUAD with late pseudotime showed high TMB and low PD-L1 expression, immune checkpoint inhibitors targeting molecules other than PD-L1 can be proposed as an appropriate immunotherapy option for tumors in progressed LUAD. Our model of the temporal evolution of the TIME and biomarkers related to immunotherapy suggested that a more precise strategy of immunotherapy could be needed according to the biological progression of lung cancer. In this respect, further study can be planned for selecting appropriate immunotherapy regimens and evaluating treatment response using pseudotime concept. Based on estimated pseudotime from transcriptomics, patients can be classified into those with early and late phase of disease. Application of different immunotherapy regimen and evaluation prognosis can be performed in each group. The further study is expected to expand usefulness and clinical significance of pseudotime in LUAD patients.

This study has clinical implication and benefits as followings. We attempted to generate simple and continuous scale of disease progression in LUAD. The genomic landscape of LUAD has been investigated in many previous studies and varying genetic characteristics were known to associate with prognosis of LUAD. It is needed to suggest more simplified and available value integrating diverse genetic information. In recent clinical filed, there are a few approaches to provide genetic profile information to LUAD patients with microarray or RNA sequencing. However, it is too complicated and difficult to deliver its clinical implication to patients. It is expected that simplified scale of disease progression is helpful to communicate with patients for discussing disease progression status, further treatment plan, and prognosis. Of course, further study is needed to construct a reference model from a larger cohort to validate and utilize pseudotime.

There are some limitations in this study. First, FDG PET examination of subjects was performed in different institutes so that there were differences in image acquisition and reconstruction methods. However, the purpose of analyzing the association between SUV and pseudotime was not to predict accurate SUV or pseudotime but to assess the overall tendency of SUV along pseudotime. Furthermore, the image acquisition protocol of each sample was not identified in the obtained clinical data. Therefore, all the data were included in a single correlation study. Further study is warranted to analyze the evolution of glucose metabolism over pseudotime more accurately using FDG PET image data from the same institute. Second, pseudotime trajectory from RNA-seq has a limitation to apply to the clinical field due to the complexity of obtaining tumor tissue and analyzing transcriptomic data from each patient. To facilitate the application of pseudotime in clinical situations, further study is underway to construct pseudotime trajectories from FDG PET images. Third, estimation of immune cell infiltration using deconvolutional method for transcriptomic data has a limitation to exactly estimate immune cell fraction of tumor tissue. In contrast, it has an advantage that quantification for large-scale dataset is available. Further study is warranted to validate actual TIME of tumor tissue with experimental methods such as immunofluorescence.

Taken together, pseudotime trajectories were successfully estimated in lung adenocarcinoma subjects from the TCGA dataset and the NSCLC radiogenomics dataset. These results show fair correlations with TNM stage, clinical outcome, and glucose metabolism, suggesting the feasibility of a new scale evaluating disease progression status. There were heterogeneous findings in the evolution of tumor immune microenvironment components over pseudotime. The present study suggested that individualized immunotherapy strategies should be selected according to different molecular characteristics evolving during disease progression.

## Data Availability Statement

RNA-sequencing data from The Cancer Genome Atlas datasets are available in National Cancer Insititute Genomic Data Commons Data Portal (https://portal.gdc.cancer.gov/). Clinical data of The Cancer Genome Atlas datasets are available in cBioPortal (https://www.cbioportal.org/). RNA-sequencing data from non-small cell lung carcinoma radiogenomics dataset (GSE103584) are available in Gene Expression Omnibus (https://www.ncbi.nlm.nih.gov/geo/).

## Author Contributions

HL and HC designed the study, performed analysis and interpretation, and wrote the manuscript. All authors contributed to the article and approved the submitted version.

## Funding

This research was supported by the National Research Foundation of Korea (NRF) and funded by the Korean government (MSIT) (No.2020M3A9B6038086, NRF-2019R1F1A1061412). This research was also funded by the Korea Medical Device Development Fund grant funded by the Korean government (Ministry of Science and ICT, Ministry of Trade, Industry and Energy, Ministry of Health & Welfare, Ministry of Food and Drug Safety) (Project Number: 202011A06).

## Conflict of Interest

HC is a co-founder and CTO of Portrai, Inc. and scientific advisor of AitheNutrigene Inc.

The remaining author declares that the research was conducted in the absence of any commercial or financial relationships that could be construed as a potential conflict of interest

## Publisher’s Note

All claims expressed in this article are solely those of the authors and do not necessarily represent those of their affiliated organizations, or those of the publisher, the editors and the reviewers. Any product that may be evaluated in this article, or claim that may be made by its manufacturer, is not guaranteed or endorsed by the publisher.
