# Supplementary figures and images for: Investigating the Clinico-Molecular and Immunological Evolution of Lung Adenocarcinoma Using Pseudotime Analysis
Source: Front Oncol. 2022 Mar 4;12:828505. doi: 10.3389/fonc.2022.828505 (PMC8931203; doi:10.3389/fonc.2022.828505)

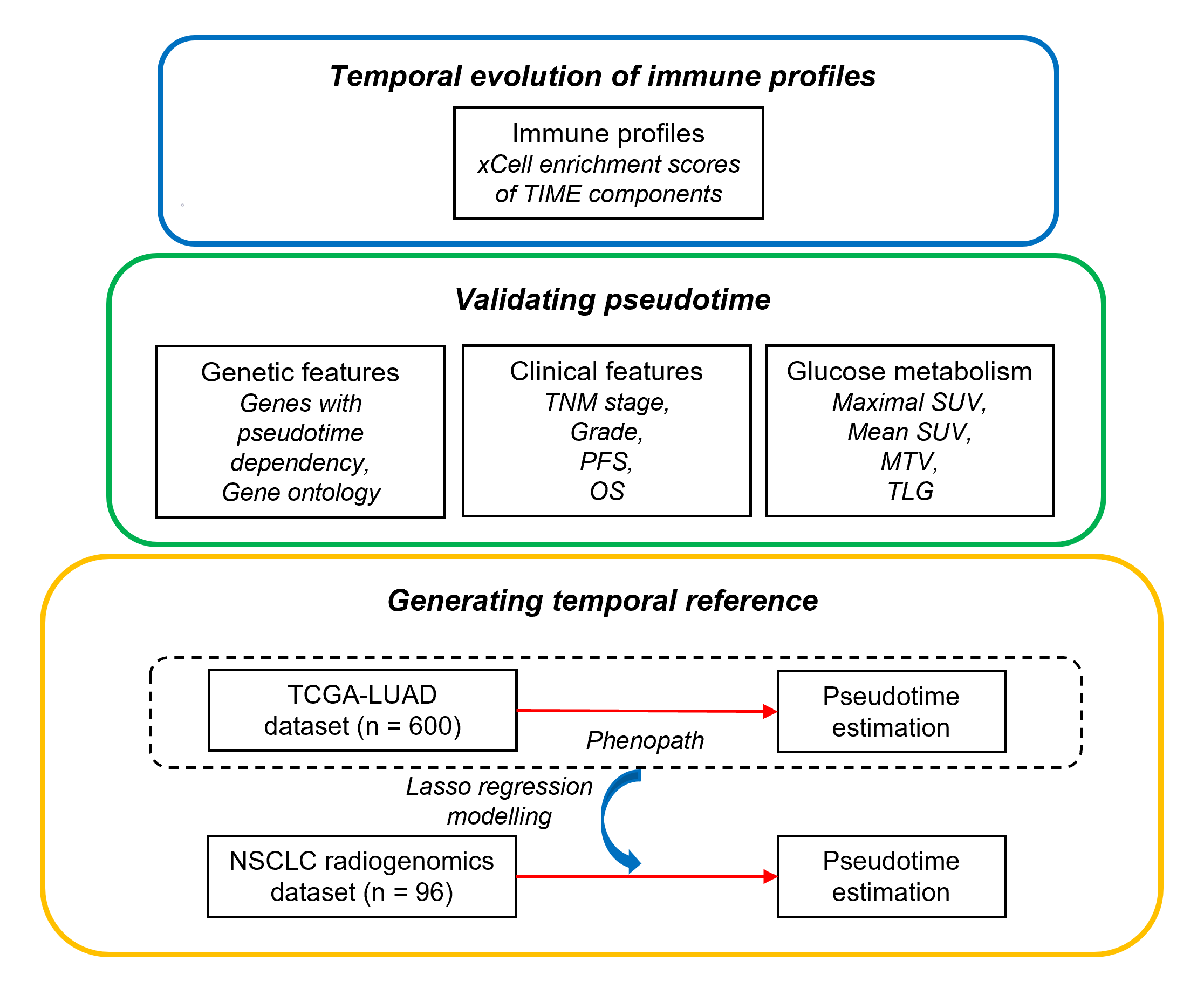

Supplement: Supplementary file 1 [file Image_1.tiff]

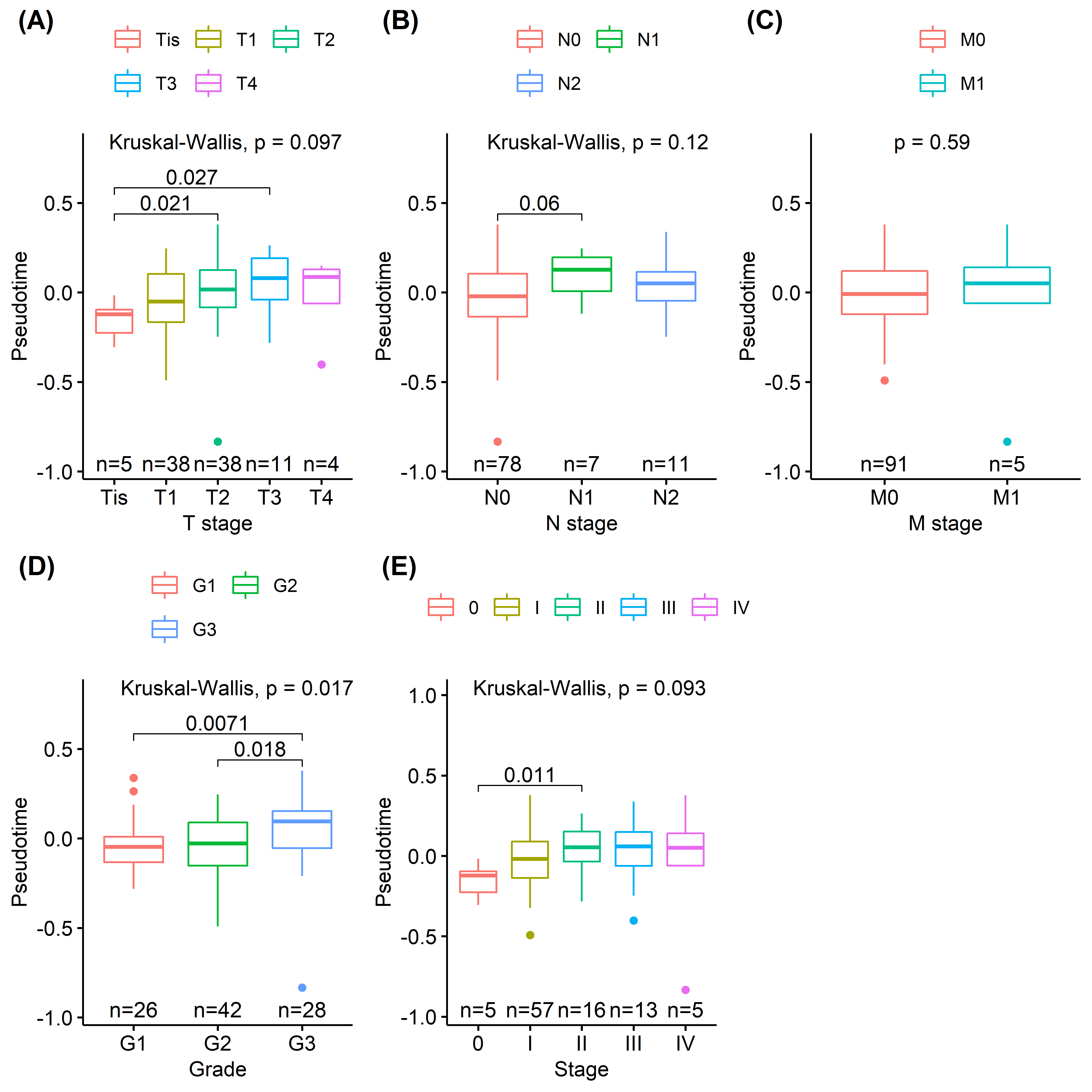

Supplement: Supplementary file 2 [file Image_2.tiff]

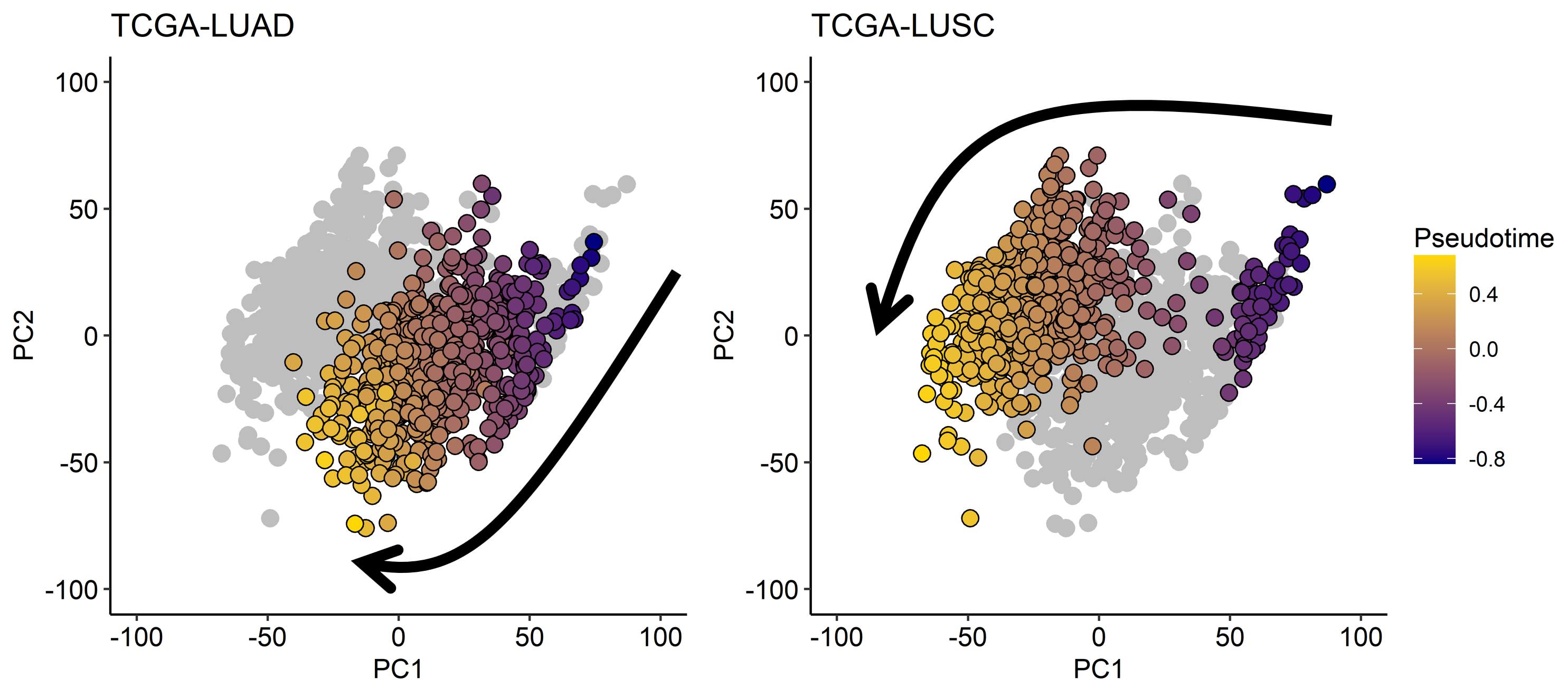

Supplement: Supplementary file 3 [file Image_3.tif]

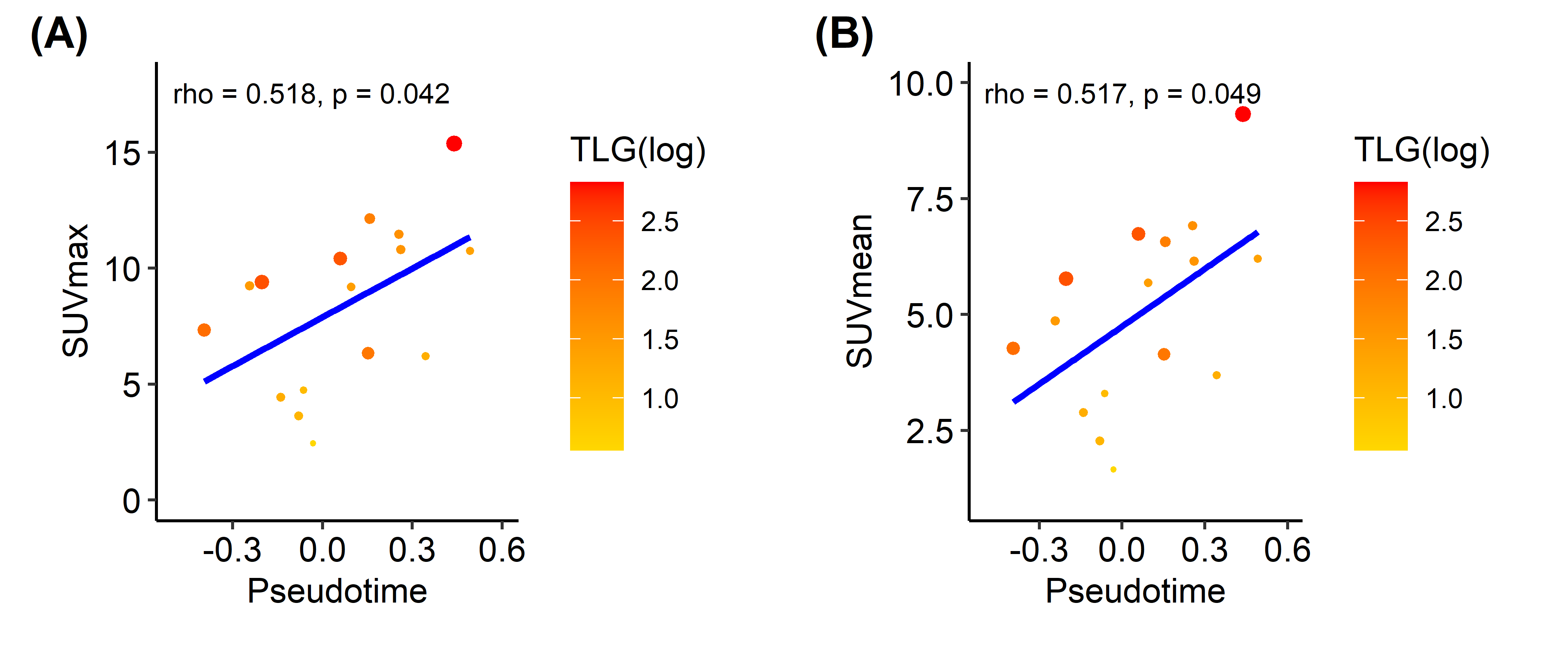

Supplement: Supplementary file 4 [file Image_4.tiff]

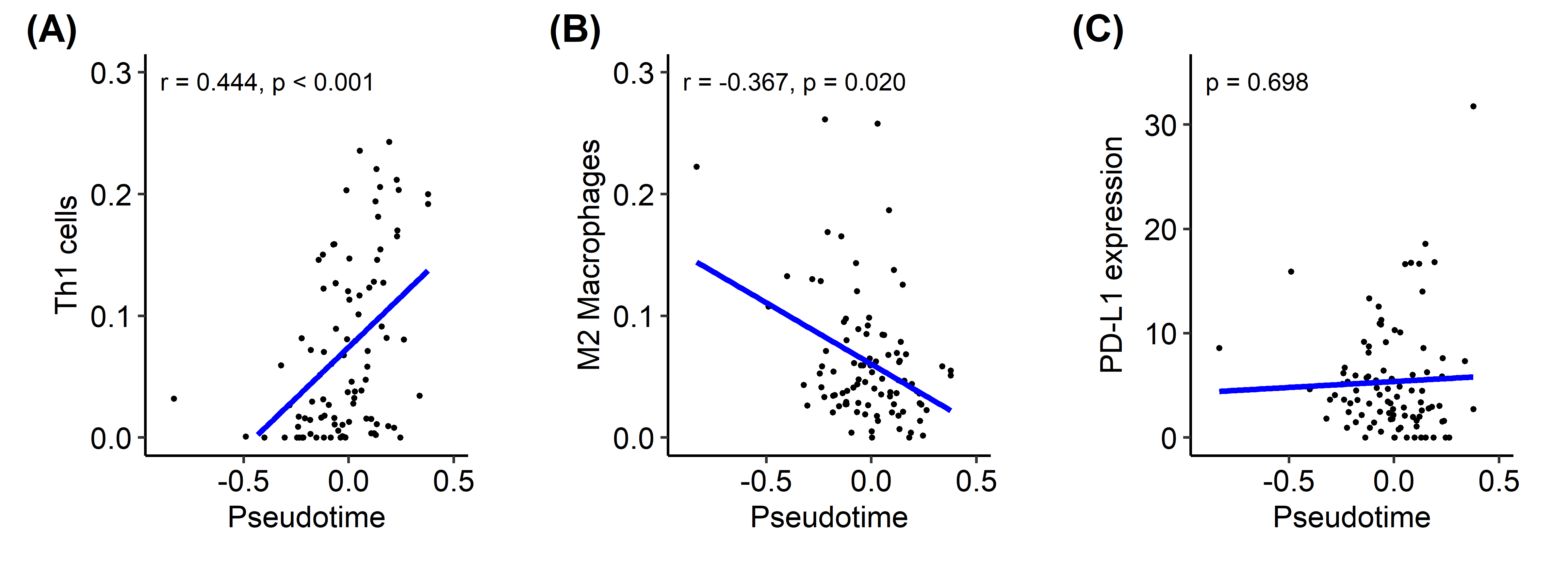

Supplement: Supplementary file 5 [file Image_5.tiff]
